# Italian validation of the Guilt And Shame Proneness Scale: preliminary results

**DOI:** 10.1192/j.eurpsy.2022.1956

**Published:** 2022-09-01

**Authors:** F. Faccini, G. Rogier, R. Cavalli, P. Velotti

**Affiliations:** 1 University of Rome Sapienza, Dynamic And Clinical Psychology And Health, Rome, Italy; 2 University of Genoa, Department Of Educational Sciences, Genoa, Italy

**Keywords:** shame, guilt, Suicide, validation

## Abstract

**Introduction:**

Background: Recently, a new instrument, the Guilt And Shame Proneness Scale has been developed and showed promising psychometric properties. However, the Italian version of the Instrument has not still been validated. In addition, despite the growing number of studies on the topic, the knowledge regarding the role played by guilt, shame and rivalry in the relationship between pathological narcissism facets and suicidal ideation.

**Objectives:**

To validate the Italian version of the Guilt And Shame Proneness Scale and to extend the knowledge regarding the relationships between guilt, shame, pathological narcissism and suicidality.

**Methods:**

We administrated, to a sample of Italian adults, the Italian versions of the GASP, the Pathological Narcissism Inventory, the Beck Suicide Inventory and the Narcissism Admiration and Rivalry Questionnaire.

**Results:**

The structural equation model testing the factorial structure of the Italian version of the GASP obtained a good fit. In addition, invariance among gender as well as other invariance tests were tested successfully. Finally, regression and mediation analyses showed that the subscale Shame Social withdraw mediate the relationship between Narcissism grandiosity and suicidal ideation. In contrast, rivalry and social withdraw in response to shame were no more predictive of suicidal ideation controlling for pathological narcissism levels.

**Conclusions:**

The Italian version of the GASP appears promising to deepen the investigation of the pathological personality topic.

**Disclosure:**

No significant relationships.

